# Agrobacterium-mediated transformation of Nicotiana glauca and Nicotiana sylvestris

**DOI:** 10.18699/VJGB-22-84

**Published:** 2022-11

**Authors:** G.V. Khafizova, T.V. Matveeva

**Affiliations:** Federal Research Center the N.I. Vavilov All-Russian Institute of Plant Genetic Resources (VIR), St. Petersburg, Russia; Saint-Petersburg State University, St. Petersburg, Russia

**Keywords:** agrobacterium-mediated transformation, regeneration, Nicotiana, агробактериальная трансформация, регенерация, Nicotiana

## Abstract

Agrobacterium-mediated transformation is the most popular approach for obtaining transgenic plants nowadays. There are plenty of protocols developed for different plant species. These protocols usually include the medium composition, the technology for preparing plant explants and cultivation conditions, as well as the choice of agrobacteria strains. Nicotiana tabacum, or cultivated tobacco, was one of the f irst successfully transformed plant species. Nicotiana tabacum is a model object in plant genetics, particularly due to its ability for transformation and regeneration. N. tabacum is a naturally transgenic plant since its genome contains a cellular T-DNA acquired from Agrobacteria. The signif icance of cT-DNA for plants has not yet been established. Some assume that cT-DNA can increase the ability of plants to regenerate due to some of the genes they contain. For example, rolC has been shown to affect the hormonal balance of plants, but the molecular mechanisms underlying this have yet to be found. RolC is also somehow involved in the secondary metabolism of plants. Like N. tabacum, Nicotiana glauca produces a wide range of secondary metabolites and contains an intact rolC gene in its genome. At the same time, unlike N. tabacum, N. glauca is a diploid species, which makes it more suitable for genetic engineering approaches. Nicotiana sylvestris is one of the ancestral species of N. tabacum and does not contain cT-DNA. The aim of this work was to develop a protocol for transformation and regeneration of N. glauca and N. sylvestris. We managed to find an optimum ratio of auxins and cytokinins that promotes both active callus formation and organogenesis in N. glauca and N. sylvestris leaf explants. The developed technique will be useful both for fundamental research that includes the N. glauca and N. sylvestris species, and for practical application in the pharmaceutical industry and biosynthesis.

## Abstract (ru)

Агробактериальная трансформация – наиболее популярный метод получения трансгенных растений. Для многих видов растений разработаны протоколы, включающие описание условий трансформации, состав питательных сред, методику подготовки растительных эксплантов и выбор штаммов агробактерий, а также соотношение растительных гормонов, необходимых для последующей регенерации эксплантов. Одним из первых успешно трансформированных видов стал культурный табак, Nicotiana tabacum, который сегодня служит модельным объектом генетики растений. Nicotiana tabacum эффективно трансформируется и легко регенерирует, что делает его удобным для генно-инженерных манипуляций. При этом N. tabacum относится к природно-трансгенным видам, поскольку содержит в своем геноме последовательности агробактериального происхождения, клеточную Т-ДНК, значение которой для растений пока не установлено. Одним из предковых видов для N. tabacum является N. sylvestris, геном которого не содержит клТ-ДНК. Предполагают, что клТ-ДНК может повышать регенерационные способности растения за счет генов, входящих в ее состав, таких как, например, rolC. Для rolC действительно показано влияние на баланс растительных гормонов, однако стоящие за этим молекулярные механизмы остаются неизвестными. Помимо участия в морфогенезе, rolC влияет на биосинтез вторичных метаболитов в растении. Вид N. glauca, как и N. tabacum, считается природно-трансгенным, несет в клТ-ДНК интактный rolC и содержит широкий спектр вторичных метаболитов. При этом, в отличие от N. tabacum, N. glauca –диплоидный вид, что делает его гораздо более удобным объектом для проведения генно-инженерных работ. Целью данной работы была разработка протокола трансформации и регенерации для видов N. glauca и N. sylvestris. На основании уже известных протоколов для других представителей рода Nicotiana нами было подобрано такое соотношение ауксинов и цитокининов, при котором листовые экспланты N. glauca и N. sylvestris переходят к активному каллусообразованию, а затем к органогенезу. С использованием разработанной методики получены трансгенные растения этих видов. Разработанная методика трансформации и регенерации полезна как для фундаментальных исследований, затрагивающих виды N. glauca и N. sylvestris, так и для практического применения в области фарминдустрии и биосинтеза.

## Introduction

Agrobacterium-mediated transformation has been the main approach for obtaining transgenic plants in laboratories for over 30 years (Sawahel, Cove, 1992). To date, transformation protocols have been developed for many plant species represented by various life forms, such as herbs, shrubs, and trees (Wang, 2015). The main differences between these protocols are determined by the choice of the agrobacterium strain, the vector, the type of plant explant and the way to prepare it for transformation. It is also necessary to select certain conditions of inoculation and co-cultivation processes, such as their duration, as well as lighting and temperature values. Due to various modifications of the protocols, it was possible to significantly increase the efficiency of agrobacterium-mediated transformation of various plant species, including economically important crops (Cheng M. et al., 2004). The process of plant regeneration that usually follows transformation also requires specific conditions for each particular species

Nicotiana tabacum is one of the first transformed species. Development of the transformation and regeneration protocols for N. tabacum led to the first transgenic tobacco plants resistant to antibiotics (Herrera-Estralla et al., 1983). To date, N. tabacum is a classic model object of plant genetics and is widely used in genetic engineering. Since N. tabacum is a valuable agricultural crop, it is well studied, and a highquality reference genome is available in the open database (Edwards et al., 2017). The N. tabacum species includes many cultivars that differ in a number of ways, including the efficiency of the regeneration process (Ali et al., 2007), which allows researchers to choose the most suitable ones for the transformation process.

The genome of N. tabacum contains DNA sequences acquired from Agrobacteria. These sequences, homologous to the agrobacterial T-DNA, are called cellular T-DNA (cT-DNA) (White et al., 1983). Plants that carry cT-DNA are considered natural transgenic species or natural genetically modified organisms (nGMOs) (Matveeva, 2018). To date, the list of nGMOs includes more than 40 genera of angiosperms (Matveeva, 2021). The function of cT-DNA for plants has yet to be established. Several hypotheses are discussed in the literature, such as increasing the adaptive capacity to arid conditions, impact on the microbial communities of the rhizosphere, enhancing regenerative abilities and resistance to subsequent agrotransformation (Chen, Otten, 2017; Matveeva, Sokornova, 2017). In addition, an increased sensitivity of natural transgenic plants to agrobacterium-mediated transformation is assumed. The experimental data obtained for different nGMO species do not add up to a unified picture. However, to date, only five natural transgenic species belonging to the genus Nicotiana have been studied for the transformation efficiency (Matveeva, Sokornova, 2017). Expanding the list of studied species may clarify this issue.

Some of cT-DNA genes have retained their activity in natural transgenic species for generations, suggesting the importance of these genes for the plant. One of these genes is rolC in the cT-DNA of N. tabacum (Chen et al., 2014) and N. glauca (Intrieri, Buiatti, 2001). The rolC gene activity is known to affect morphogenetic processes, as well as the secondary metabolism of plants, although the molecular mechanisms underlying its effects have not yet been elucidated (Khafizova, Matveeva, 2021). For studying gene activity, various approaches can be used. Silencing and controlled gene activation are the most popular among them. However, they require developed methods of transformation and regeneration under in vitro conditions for specific plant species

This work is devoted to the development of transformation and regeneration methods for the N. glauca and N. sylvestris species. N. glauca, like N. tabacum, is a natural transgenic plant carrying an intact rolC in its cT-DNA. And, like N. tabacum, it contains a wide range of secondary metabolites (Long et al., 2016). At the same time, N. glauca is a diploid, which makes it a much more convenient object for genetic engineering manipulations comparing to allotetraploid N. tabacum. N. sylvestris is one of the ancestral species of N. tabacum and its genome does not contain cT-DNA (Yukawa et al., 2006). Like N. glauca, N. sylvestris is a diploid species

Two plasmids were constructed in this work: the first contains rolC under an inducible promoter to create N. sylvestris plants with controlled expression of rolC. The second plasmid contains a CRISPR/Cas9 cassette with 2 guide RNAs targeting rolC aiming to “turn off” this gene in N. glauca. However, protocols for the transformation and regeneration of N. sylvestris and N. glauca have not previously been developed. Therefore, it was necessary to design such protocols based on existing ones. The possibility to create transgenic N. sylvestris and N. glauca plants will expand the range of research involving these species. For example, N. glauca mutants for various genes in biosynthesis pathways will contribute to the study of the molecular mechanisms of secondary metabolism. N. glauca plants with inactivated rolC and N. sylvestris plants carrying rolC, obtained in this work, will be further used to investigate the functions of the rolC gene, contributing to fundamental studies in horizontal gene transfer from agrobacteria to plants

## Materials and methods

Aseptic plants Nicotiana glauca (var. 359 from the Federal state budget scientific institution “All-Russian Scientific Research Institute of Tobacco, Makhorka and Tobacco Products” collection) and Nicotiana sylvestris (obtained from the Federal state budget scientific institution “All-Russian Scientific Research Institute of Tobacco, Makhorka and Tobacco Products” collection) were used in this work. Plants were grown in vitro and maintained by cuttings on Murashige–Skoog (MS) medium (Murashige, Skoog, 1962) with 20 g/L sucrose at 23 °C and a photoperiod of 16 hours day/8 hours night.

For the transformation of N. glauca plants, a pHSE401_roC vector was prepared. It contained a cassette for the rolC gene editing: 2 guide RNAs and Cas9 under the control of CaMV 35S; as well as kanamycin and hygromycin resistance genes. For the transformation of N. sylvestris plants, the pB7WG2D_ PdexA4rolC vector was prepared. The pB7WG2D_ PdexA4rolC vector contained the rolC gene sequence from A. rhizogenes under a dexamethasone-inducible promoter along with spectinomycin and glufosinate resistance genes.

Vector design. The sequence of the rolC gene and the dexamethasone-inducible promoter was obtained from transgenic plants previously created by colleagues (Mohajjel-Shoja et al., 2011). PCR was carried out in a volume of 20 μL using DreamTaq PCR master mix (Thermo Scientific) according to the prescription into a Tertsik amplifier (DNA-technology) by the following program: 95 °C – 5 minutes, 40 cycles (95 °C – 20 sec, 60 °C – 30 sec, 72 °C – 90 sec), 72 °C – 5 minutes. To obtain the sequence “promoter + rolC ”, the following primers were used, DexF: CGCTACTCTCCCAAAACCAA, DexR: GGCCAGTGAATTCTCGACTC. Primers were synthesized by Evrogen. The resulting sequence was placed into the pENTR/D-TOPO cloning vector (https://www.addgene.org/ vector-database/2519/), which was used to transform E. coli Top10 strain. Bacteria grew on LB medium with kanamycin (100 mg/L) at 37 °C for 14 hours. Isolation of plasmid DNA from the resulting colonies was carried out using a Plasmid Miniprep Kit (Evrogen). PCR with primers DexF and DexR was performed to detect the insertion of rolC with an inducible promoter. The insert was then cloned into the destination vector pB7WG2D (https://gatewayvectors.vib.be/collection/ pb7wg2d) using the Gateway system (Invitrogen, USA). The resulting plasmids were tested by PCR with primers DexF and DexR. After plasmid verification, the Agrobacterium EHA105 strain was transformed with pB7WG2D_PdexA4rolC

The pHSE401_roC vector was created using the pHSE401 plasmid (https://www.addgene.org/62201/), according to the protocol described by Xing (Xing et al., 2014). The pHSE401 vector was kindly provided by the senior researcher of the Department of Genetics and Biotechnology, St. Petersburg State University, Tvorogova V.E. The NgrolC gene (Acs. X03432.1; 145–687) was chosen as a target. The selection of 19-nt target sequences and the final verification of the vector by PCR and restriction methods were carried out according to the protocols described by Xing (Xing et al., 2014). After verification, pHSE401_roC was used to transform the Agrobacterium strain AGL1.

Plants transformation. For plant transformation, overnight cultures of agrobacteria were prepared. Young leaves (3–4 upper leaves) were selected from aseptic plants in laminar box. Along the perimeter of the leaf blade, incisions 2–3 mm long were made with a sterile scalpel. The cuts crossed the leaf vein. The leaves were then placed in a mixture of liquid Murashige–Skoog medium (MS without agar) and overnight bacteria culture in a ratio of 1:1 for 2 hours. At the end of cultivation, the liquid from the leaves surfaces was removed with sterile filter paper, and the leaves were transferred to a solid MS medium.

Plants regeneration. Plates with leaf explants were kept for 2 days at 23 °C in the dark. Then the explants were transferred to MS medium containing 250 mg/L of cefotaxime, 2 mg/L of 6-benzylaminopurine (BAP), and 1 mg/L of naphthylacetic acid (NAA). Every 8–10 days the explants were transplanted onto a fresh medium containing hormones and an antibiotic. After the formation of organogenic calli and the initiation of shoot formation (4–6 weeks from the moment of transformation), the calli were placed on a hormone-free MS medium containing an antibiotic. The grown shoots were separated from the calli and placed on the MS medium with a mixture of antibiotics: 50 mg/L of cefotaxime, 10 mg/L of a selective antibiotic. Cefotaxime was used to kill agrobacteria. A selective antibiotic was used to select transformants.

Hygromycin was used for N. glauca because pHSE401_roC contains the HygR gene providing hygromycin resistance. For N. sylvestris, glufosinate was used as a selective antibiotic, since the pB7WG2D_PdexA4rolC vector contains the BAR gene.

Transformants analysis. Those shoots that remained green on the selecting medium were checked by PCR for the presence of a transgenic insert. DNA was isolated using the CTAB method (Murray, Thompson, 1980). For N. glauca, PCR analysis was carried out with primers pHSE401RolCF ( 5 ′ T G T C C C A G G AT TA G A AT G AT TA G G C ) a n d pHSE401RolCR (5′AGCCCTCTTCTTTCGATCCATC AAC) to a CRISPR cassette. PCR was performed in a volume of 20 μL using DreamTaq PCR master mix (Thermo Scientific) according to the prescription in the Tertsik amplifier (DNA-technology) by the following program: 95 °С – 5 minutes, 40 cycles (95 °С – 10 sec, 58 °С – 30 sec, 72 °С – 30 sec), 72 °С – 5 minutes. The amplicons were visualized and separated on a 1 % agarose gel in TAE buffer.

Validation for N. sylvestris was performed by real-time PCR with primers for the BAR gene contained in the vector (BarF: AGCCCGATGACAGCGACCAC; BarR: CGCCGAT GACGCGGGACAA). The DNA of the transgenic N. tabacum plant containing the rolC gene was used as a positive control. A sample without DNA was used as a negative control. PCR was performed in a volume of 20 μL using Fast SYBR Green master mix (Thermo Scientific) according to the prescription in ANK-32-M amplifier (Synthol) by the following program: 95 °С – 5 minutes, 40 cycles (95 °С – 10 sec, 58 °С – 30 sec, 72 °С – 30 sec), 72 °С – 5 minutes. Primers were synthesized by Evrogen.

## Results

**Fig. 1.**
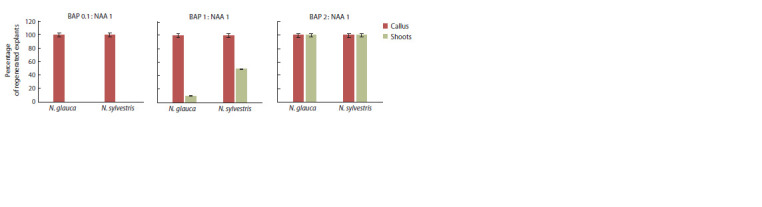
Results of a preliminary experiment on the regeneration of N. sylvestris and N. glauca leaf explants.

Transformation and regeneration protocols for N. sylvestris and N. glauca were developed by optimization of existing protocols for different Nicotiana species. We conducted a preliminary experiment to evaluate the regeneration efficiency of N. sylvestris and N. glauca explants. The explants were put on media with different hormone ratios: 2 mg/L BAP and 1 mg/L NAA, 1 mg/L BAP and 1 mg/L NAA, 0.1 mg/L BAP and 1 mg/L NAA. At first, all explants actively formed callus, no differences were observed on various media at this stage. However, upon transition to organogenesis, the amount of cytokines began to affect the efficiency of regeneration (Fig. 1). While 100 % of explants of both N. sylvestris and N. glauca formed shoots on a medium with a high content of cytokinins, only 50 % of explants of N. sylvestris and 10 % of N. glauca switched to organogenesis on 1 mg/L BAP and 1 mg/L NAA medium. Shoots did not develop on the medium 0.1 mg/L BAP and 1 mg/L NAA.

Thus, in a preliminary experiment, we noted active regeneration and shoot formation processes on leaf explants when 2 mg/L BAP and 1 mg/L NAA were added to the medium. For comparison, the traditional medium for the induction of callus formation in N. tabacum contains 0.5 mg/L BAP and 2 mg/L NAA (Draper et al., 1991), and the medium for N. benthamiana contains 1 mg/L BAP and 0.1 mg/L NAA (Hasan et al., 2014). Analyzing the literature, we also noticed different ways of preparing explants: in the classic version of “leaf disk transformation” cut out fragments of the leaf blade that do not contain veins are used (Wang, 2015). There are also options for cutting the leaf blade into pieces (Draper et al., 1991) and the deep vein incision method. We used the cutting method and the deep vein incision method in the preliminary experiment. The way of the explant preparation did not affect the efficiency of transformation.

**Fig. 2.**
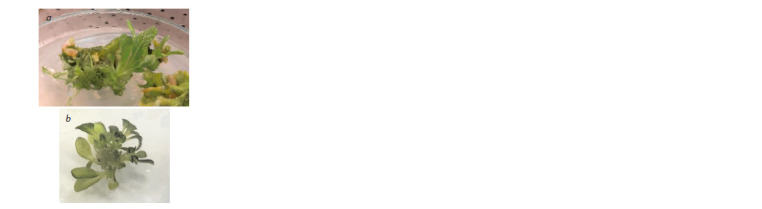
Regeneration of shoots from leaf explants after co-cultivation with agrobacteria, N. sylvestris (a) and N. glauca (b).

Using the developed protocols we performed the transformation of N. sylvestris and N. glauca leaf explants, 500 for each species. As a result, regenerated plants were obtained from 498 N. sylvestris explants and 491 N. glauca explants (Fig. 2). Several explants (2 and 9, respectively) were contaminated during transplantation and removed from the experiment. Thus, the results obtained on a large sample are consistent with the results of the preliminary experiment.

**Fig. 3.**
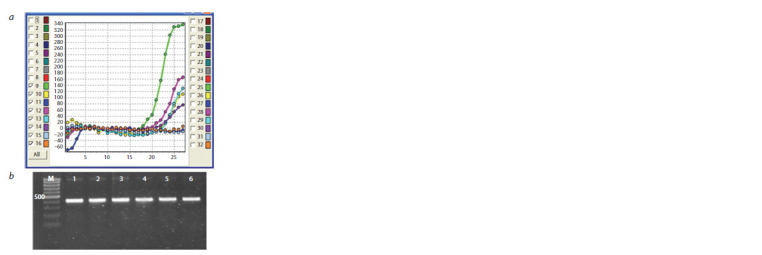
Confirmation of transgenic inserts in regenerated plants: a, N. sylvestris (9 – positive control, 10–15 – regenerated plants, 16 – negative control); b, N. glauca (1–6 – regenerated plants)

The shoots formed on the medium with BAP and NAA were transplanted onto the medium with a selective antibiotic. For N. sylvestris, 15 plants were obtained on the medium with glufosinate, and 12 plants for N. glauca on the medium with hygromycin, which is 3 and 2.4 % of the regenerated explants. Plants that remained green and rooted on the selective medium were tested for the presence of the transgenic insert. Positive results were obtained for all N. glauca regenerants and 12 N. sylvestris regenerants (Fig. 3). For N. sylvestris, real-time PCR was used to detect a signal in transgenic plants, indicating the presence of the BAR gene contained in the vector (see Fig. 3, a). In the case of N. glauca, 423 bp sequences corresponding to the fragment of the CRISPR cassette were obtained (see Fig. 3, b).

Therefore, N. sylvestris plants with the rolC gene under a dexamethasone-inducible promoter were created, as well as N. glauca plants carrying a CRISPR cassette for “turning off” the rolC gene. Currently, some plants are planted in a greenhouse and some are grown in vitro. Next, they will be used to study the function of the rolC gene by controlled activation of its expression in N. sylvestris and by gene silencing in N. glauca.

Agrobacterium-mediated transformation and regeneration protocols for N. sylvestris and N. glauca were developed in this study. We have shown transformation efficiencies of 3 and 2.4 % for these species, respectively. The efficiency can be increased by adding acetosyringone to the medium. However, the described protocol is sufficient to obtain transgenic N. sylvestris and N. glauca plants, which was successfully demonstrated in this work.

## Discussion

Agrobacterium-mediated transformation is the most common way to obtain transgenic plants today. For many plant species, protocols for transformation and regeneration have been developed (Wang, 2015). While some species regenerate easily through the stages of organogenic callus and shoot development, other species show low regeneration efficiency. For example, in Pisum sativum, less than half of the somatic embryos develop into plants (Loiseau et al., 1995).

Many Nicotiana species regenerate easily and can also be grouped according to their tendency to shoot formation or root formation when regenerating (Matveeva, Sokornova, 2017). N. tabacum actively forms both roots and shoots. At the same time its ability to form callus on media with different ratios of hormones is of particular interest. There are protocolos describing N. tabacum callus formation on both auxin-dominated media (Draper et al., 1991; Ali et al., 2007) and cytokinin-dominated media (Horsch et al., 1985; Otten, Helfer, 2001). Researchers note the active formation of calli on explants, regardless of the protocol chosen. A similar picture is shown for N. rustica, which equally forms both roots and shoots during regeneration (Gill et al., 1979; Furze et al., 1987; Tinland et al., 1992). For other Nicotiana species, this feature has not been noted in the literature

Cellular T-DNA was named as a possible explanation of the increased ability to regenerate, since T-DNA contains genes that affect the plant hormonal balance (Ichikawa et al., 1990). At the same time, those genes, which are called plast genes, differ in their effects (Otten, 2018). Therefore, it is important which plast genes carry cT-DNA and whether their reading frames remain intact. The N. tabacum genome holds three cT-DNAs of different composition, containing plast genes with intact frames (Chen et al., 2014). However, the N. rustica genome lacks cT-DNA (Intrieri, Buiatti, 2001). Our results also refute this hypothesis. Likewise, we did not confirm the assumption about the increased sensitivity of natural transgenic species to agrotransformation. The transformation efficiency turned out to be about the same for N. glauca containing cT-DNA and for N. sylvestris that does not contain cT-DNA in the genome. A similar trend was noted at the stages of callusogenesis and subsequent organogenesis. While N. glauca contains an intact rolC gene, which affects the balance of cytokinins in the plant (Schmulling et al., 1988), and N. sylvestris does not contain cT-DNA (Intrieri, Buiatti, 2001), regeneration in these species is triggered by the same ratio of hormones.

At the same time, differences in the efficiency of regeneration were noted at the intraspecific level. For example, N. tabacum cultivar SPTG-172 regenerates better on a medium containing 0.2 mg/L BAP and 2 mg/L NAA, while for cultivar K-399, the ratio of 0.2 mg/L BAP and 1 mg/L NAA is preferable. But even on a more suitable medium, K-399 forms less callus and shoots than SPTG-172 (Ali et al., 2007). Ali and colleagues explain such differences by the genotype influence. The effect of genotype on callusogenesis and organogenesis has already been shown for peas (Lutova et al., 1994; Saschenko, 2014) and cruciferous plants (Ockendon, Sutherland, 1987; Narasimhulu, Chopra, 1988). Particular qualities of the genotype, which determine the response to the medium and cultivation conditions, are often called the main factor that affect the regeneration efficiency (Pang et al., 2000). Ali and colleagues also noted that auxin-rich media are preferred for some tobacco cultivars, while cytokinin-rich media are preferable for others (Ali et al., 2007). In order to confirm or refute this hypothesis, it is necessary to conduct a study on a larger number of N. tabacum cultivars.

Despite a significant number of developed protocols for regeneration and studies on this topic (Wang, 2015), the genetic mechanism responsible for morphogenetic reactions remains unknown. In an attempt to establish it, geneticists and biochemists are actively studying both the biosynthesis pathways of plant hormones and their signaling, as well as the mutual influence of various hormones on regeneration processes in different plant species (Su, Zhang, 2014). More and more specific points of hormones interaction are being identified. For example, it has been shown that a participant in the auxin signaling pathway ARF3 (AUXIN RESPONSE FACTOR3) directly suppresses cytokinin biosynthesis during shoot regeneration by binding the AtIPT5 gene promoter (Cheng Z.J. et al., 2013). However, the questions of why some plants regenerate more easily than the others, and what factors directly affect these processes, have yet to be answered.

The techniques we have developed for N. glauca and N. sylvestris expand our knowledge of the regeneration conditions of various Nicotiana species. In the case of N. glauca, this technique can contribute to the development of the pharmaceutical industry, since it allows to create various mutants for studying the biosynthesis of secondary metabolites. Both the transgenic N. glauca and N. sylvestris created in this work will advance fundamental studies of horizontal gene transfer. Despite the fact that the species N. glauca and N. sylvestris are not closely related (Clarkson et al., 2004), the same ratio of hormones triggers the induction of callusogenesis and regeneration. In this regard, the proposed technique can become a starting point for the development of new protocols based on it, as was described in this article

## Conclusion

A technique for agrobacterium-mediated transformation and effective regeneration of N. glauca and N. sylvestris species has been developed. On its basis, it is possible to create protocols for other Nicotiana species by varying the ratio of the main exogenous plant hormones added to the medium. The technique includes agrotransformation of plant explants using the leaf disk method, followed by cultivation on MS nutrient medium containing antibiotics as well as plant hormones in the amount of 2 mg/L BAP and 1 mg/L NAA. The choice of antibiotic is determined by the resistance genes in the vector used for the transformation. On this medium, leaf explants of N. glauca and N. sylvestris actively regenerate with the formation of first callus mass, and then with the development of shoots. The designed protocol will be useful both for fundamental research involving N. glauca and N. sylvestris species and in practical areas. For example, to study the phenomenon of horizontal gene transfer and related changes in the plant genome, as well as in the researches related to the biosynthesis of various metabolites, a wide range of which is synthesized in N. glauca.

## Conflict of interest

The authors declare no conflict of interest.
